# Thermal Time Model for Egyptian Broomrape (*Phelipanche aegyptiaca*) Parasitism Dynamics in Carrot (*Daucus carota* L.): Field Validation

**DOI:** 10.3389/fpls.2016.01807

**Published:** 2016-12-12

**Authors:** Amnon Cochavi, Baruch Rubin, Guy Achdari, Hanan Eizenberg

**Affiliations:** ^1^Department of Phytopathology and Weed Research, Newe Ya'ar Research Center, Agricultural Research OrganizationRamat Yishay, Israel; ^2^R. H. Smith Institute of Plant Sciences and Genetics in Agriculture, Faculty of Agricultural, Food and Environmental Sciences, The Hebrew University of JerusalemRehovot, Israel

**Keywords:** broomrape, growing degree days model, beta function, sigmoid curve, cross validation

## Abstract

Carrot, a highly profitable crop in Israel, is severely damaged by *Phelipanche aegyptiaca* parasitism. Herbicides can effectively control the parasite and prevent damage, but for optimal results, knowledge about the soil–subsurface phenological stage of the parasite is essential. Parasitism dynamics models have been successfully developed for the parasites *P. aegyptiaca, Orobanche cumana*, and *Orobanche minor* in the summer crops, tomato, sunflower, and red clover, respectively. However, these models, which are based on a linear relationship between thermal time and the parasitism dynamics, may not necessarily be directly applicable to the *P. aegyptiaca*–carrot system. The objective of the current study was to develop a thermal time model to predict the effect of *P. aegyptiaca* parasitism dynamics on carrot growth. For development and validation of the models, data was collected from a temperature-controlled growth experiment and from 13 plots naturally infested with *P. aegyptiaca* in commercial carrot fields. Our results revealed that *P. aegyptiaca* development is related to soil temperature. Moreover, unlike *P. aegyptiaca* parasitism in sunflower and tomato, which could be predicted both a linear model, *P. aegyptiaca* parasitism dynamics on carrot roots required a nonlinear model, due to the wider range of growth temperatures of both the carrot and the parasite. Hence, two different nonlinear models were developed for optimizing the prediction of *P. aegyptiaca* parasitism dynamics. Both models, a beta function model and combined model composed of a beta function and a sigmoid curve, were able to predict first *P. aegyptiaca* attachment. However, overall *P. aegyptiaca* dynamics was described more accurately by the combined model (RMSE = 14.58 and 10.79, respectively). The results of this study will complement previous studies on *P. aegyptiaca* management by herbicides to facilitate optimal carrot growth and handling in fields infested with *P. aegyptiaca*.

## Introduction

Carrot (*Daucus carota*, Apiaceae) is widely grown throughout Europe and the Mediterranean area, including Israel, where it has become a high-income cash crop. In the Mediterranean area, where carrot is grown all the year round, two species of broomrape, *Orobanche crenata* and *Phelipanche aegyptiaca*, which are chlorophyll-deficient root holoparasites, parasitize the carrot taproot, and roots (Parker, 2012). In highly infested fields, broomrape can cause a total yield loss (Bernhard et al., 1998). Although both species parasitize carrot, there are some differences between them, such as host range and base temperature for germination (Kebreab and Murdoch, 1999), with the germination temperature being crucial for parasite development and affecting the parasite's ability to attach to the crop species. *O. crenata* also parasitizes plants of Fabaceae family, while *P. aegyptiaca* also parasitizes plants of the Solanaceae family (Heide-Jørgensen, 2013). Since many Solanaceae crops are grown in the Mediterranean area, *P. aegyptiaca* is widely spread in this area.

Chemical control has been found to be effective for managing broomrape damage to crops (Eizenberg et al., 2012a). The various application methods that are in widespread practice differ mainly in terms of the broomrape developmental stage at which they are applied. In one of the most commonly used methods, the herbicide is applied during the broomrape seed germination stage with the aim to prevent elongation of the parasite haustorium; for example, for tomato, sulfosulfuron is applied to prevent haustorium elongation in *P. aegyptiaca* seeds (Eizenberg et al., 2012a). In this method, the herbicide is applied to the crop foliage but is immediately washed to the soil by sprinkler irrigation. In another, more complicated method, the underlying idea is that the sprayed herbicide must move through the host plant vessels to reach the parasite tubercle after the parasite has established a connection with the host xylem and phloem system. Since broomrape is a strong sink, absorbing water and nutrients from the host, the herbicide will indeed move rapidly through the plant vascular system to the parasite (Eizenberg et al., 2012b). In view of these application methods, there is a need to establish the optimal conditions for effective and efficient broomrape control without causing damage to the host. A previous study has shown that control of *P. aegyptiaca* growing on carrots can be achieved by application of low doses of glyphosate to the carrot foliage (Jacobsohn and Kelman, 1980). The findings of that study were confirmed by recent studies in Israel that demonstrated effective glyphosate control of *P. aegyptiaca* parasitism at different infestation levels under different climate regimes (Cochavi et al., 2015, 2016).

As discussed above, both methods of control require knowledge of the broomrape developmental stage, with correct timing of herbicide application being crucial for effective broomrape control. For example, if a systemic herbicide is applied before the broomrape seedlings have attached to the host roots, the parasite will not be controlled, and if the herbicide is applied after *P. aegyptiaca* biomass has accumulated, herbicide efficiency will be reduced (Eizenberg et al., 2006). The importance of timing was illustrated by Castejon-Muñoz et al. (1993), who found that bringing forward the sowing dates of sunflower (*Helianthus annuus*) seeds reduced *Orobanche cernua* parasitism and yield loss (Castejon-Muñoz et al., 1993). Another factor having a significant influence on the broomrape developmental stage—and hence on the herbicide application regime—is temperature. Kebreab and Murdoch (1999), for example, found that four different broomrape species germinated in different temperature ranges. They showed that for *O. crenata* the highest germination rate of 70% was obtained at 15–20°C, whereas for *P. aegyptiaca* seeds almost 100% germination was obtained at temperatures ranging between 15 and 35°C (Kebreab and Murdoch, 1999). The base temperature for development—a critical factor affecting parasitism—is particularly important in models for herbicide application, as we will elaborate in detail below. In such models, the base temperature is taken as the lower threshold below which there is no development of the parasite. For instance, Ephrath et al. (2012) defined 10°C as the minimal temperature for development of *P. aegyptiaca* on tomato roots. Murdoch and Kebreab (2013) demonstrated that germination of *Orobanche* seeds cannot occur below a temperature of 4.9°C. Mesa-Garcia and Garcia-Torres found that *O. crenata* develops faster on broad bean roots under low winter temperatures (Mesa-García and García-Torres, 1986). However, unlike *O. crenata, P. aegyptiaca* parasitizes crops the year over at all temperatures.

In the Northern hemisphere, the carrot growing season extends for 120–180 days after sowing. The season can start in July (summer) and end in January (winter) or start in December (winter) and end in May (summer). Therefore, carrots are grown under hugely disparate temperature regimes, with temperatures ranging from winter lows falling to 0°C to extremely high summer temperatures of above 35°C. Finch-Savage et al. (1998) found that the minimal temperature for carrot seed germination and seedling emergence is 2°C. Carrots sown between April and July (spring/summer) are not parasitized by *O. crenata* and *P. aegyptiaca*, but the main sowing season is during fall and winter, which gives maximal yields: it was found that even when plants were infested, the yield was higher than that for non-infested plants grown through the summer (Eizenberg et al., 2001).

To determine the optimal protocol for precise and effective herbicide application for broomrape control that takes into account factors such as those described above, a number of studies have focused on developing prediction models based on thermal time as measured in growing degree days (GDDs). Some studies have described a positive correlation between soil temperature and broomrape parasitism on different species. Ephrath et al. (2012), for example, found a positive linear correlation between increased temperatures and *P. aegyptiaca* development on tomato roots. A similar correlation was described between *Orobanche minor* and *Orobanche cumana* parasitism on red clover and sunflower roots, respectively (Eizenberg et al., 2005a, 2012b). According to these models, decision support systems for effective parasite management were developed for each crop separately. There is, however, no prediction model for correlation between soil temperature and *P. aegyptiaca* parasitism in carrot. The objective of the current study was thus to develop a robust thermal time model for the quantification and prediction of the parasitism dynamics of *P. aegyptiaca* in carrot.

## Materials and methods

### Plant material

Carrot cv. “Nairobi” (Bejo Seeds, Oceano, CA, USA) was used in all experiments. *Phelipanche aegyptiaca* inflorescences were collected in 2008 from a broomrape-infested tomato field (Mevo Hama, Israel). Seeds were sieved through a 300-mesh sieve and stored in the dark at 4°C until use. To determine the germination potential of broomrape seeds at the beginning of the experiments, a germination test was performed under standard conditions at 25°C with the standard synthetic stimulant GR24 (Yoneyama et al., 2013). GR24 was applied at a concentration of 10 g·kg^−1^ soil (10 ppm) after 12 days of pre-conditioning, resulting in a germination rate of 84%. Seeds of the same lot were used for soil infestation in controlled environment experiments and for planting above transparent tubes in the field experiments for monitoring *in-situ* parasitism using a minirhizotron, according to Eizenberg et al. (2005b).

### Controlled-environment experiment

Carrot seeds were sown in 2-L pots (two seeds per pot) in infested (15 mg·g^−1^ soil) and non-infested Newe Ya'ar soil [Chromic Haploxerert (a fine-clayey, montmorillonitic, thermic soil; 55% clay, 25% silt, and 20% sand, 2% organic matter, pH 7.2)]. Plants were grown under different temperatures regimes (16/10, 22/16, 28/22, and 34/28°C day/night) and different day lengths (16 h/8 h and 8 h/16 h day/night); each treatment included five replicates. At the end of the experiment, after 150 days, emerged broomrape inflorescences were counted, and biomass was measured. Thereafter, carrot taproots were removed from the pots and washed free of soil for biomass measurement. In addition, parasite total biomass, including weight of the inflorescences, was measured.

### Field observations

The field trials were performed over the years 2009–2012. With the aim to collect wide time and space temperature range patterns, 13 field trials were conducted in commercial carrot fields in Israel (Table 1). At each location, four transparent minirhizotron tubes were placed in the soil according to Eizenberg et al. (2005b). Ten carrot seeds were sown in the soil above each tube and germinated together with the seeds in the entire field. Once a week from carrot germination until broomrape emergence, broomrape parasitism in the soil sub-surface for each tube was monitored using a minirhizotron camera (Bartz Technology, Carpinteria, CA, USA). Hourly soil temperature at a depth of 10 cm was recorded with temperature data logger (Onset®, Hobo data loggers, Cape Cod, MA, USA).

**Table 1:** **Details of field experiments conducted between 2009 and 2012 in 13 locations in Israel**.

**Location**	**Sowing date**	**Days to appearance**	**Average temperature (°C)**	**Max. temperature (°C)**	**Min. temperature (°C)**
1	17/11/2009	41	15.39	28.26	11.63
2	10/12/2010	61	13.35	26.49	5.14
3	13/10/2010	36	21.53	32.39	14.23
4	10/11/2010	42	16.9	27.37	8.18
5	21/12/2010	63	13.48	21.47	0.00
6	20/09/2011	31	21.9	28.56	16.43
7	10/08/2011	39	28.84	46	23.39
8	06/10/2011	38	19.22	26.88	12.01
9	26/10/2011	55	14.23	28.85	4.73
10	14/11/2011	63	12.75	23.1	0.01
11	30/11/2011	69	11.76	21	4.10
12	02/09/2010	40	27.70	36.4	20.23
13	25/07/2012	33	27.91	36.08	22.33

To optimize temperature measurements, soil temperature values were summed in two methodologies: (a) daily average temperature calculated as the average of the daily minimum and maximum temperatures minus the base temperature; (b) hourly measurements summed by their relative parts, i.e., each measurement was divided by 24.

### Broomrape appearance and development analysis

**Figure 1:**
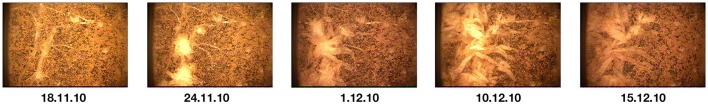
**Detection of ***Phelipanche aegyptiaca*** tubercles with a minirhizotron camera**.

Minirhizothron images were analyzed to determine the number of broomrape tubercles formed on each tube. First tubercle attachment was defined as the first image in which a small tubercle could be seen. To prevent false identification, the determination was confirmed by identifying the same tubercle in the next session a week later. The number of broomrape tubercles was considered as maximal when no additional attachments were observed (Figure 1). To obtain the partial development value of the parasite, the number of tubercles recorded in each session was divided by the maximal number of observed tubercles on the specific tube (Eizenberg et al., 2005b).

### Models for predicting *P. aegyptiaca* development dynamics

Data for *P. aegyptiaca* development and soil temperatures at each location were collected using the minirhizotron system. Several GDD models were tested with the aim to find the correlation between soil temperature and broomrape appearance, as described below.

#### Linear model

A linear model was developed according to Eizenberg et al. (2005a):

(1) $$\begin{array}{l} T_{daily}=\frac{T_{max}+T_{min}}{2}-T_{base} \end{array}$$

where *T*_*daily*_ is the calculated temperature, *T*_*max*_ and *T*_*min*_ are the daily maximum and minimum temperatures, respectively, and *T*_*base*_ is the minimum temperature for development (in carrot 2°C; Finch-Savage et al., 1998). Daily GDDs were summed to the limit value:

(2) $$\begin{array}{l} GDD_{limit}=\sum_{i=1}^{n}T_{n} \end{array}$$

The model predicts that higher temperatures accelerate the development rate.

#### Beta-function model

**Figure 2:**
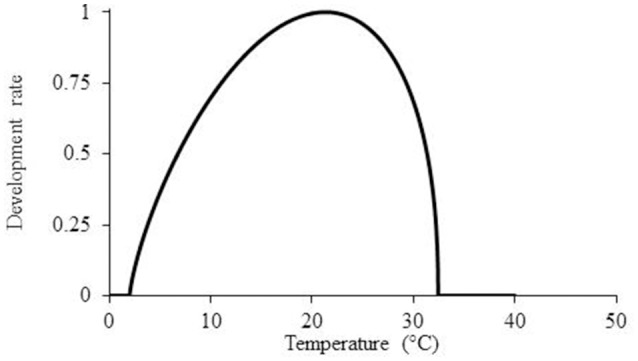
**Mathematical model for the description of ***P. aegyptiaca*** development rate as temperatures rise according to beta function model**.

A four-parameter beta-function equation was used to calculate the effect of temperature on the parasitism dynamics:

(3) $$\begin{array}{l} r={\left(\left(\frac{T-T_{b}}{T_{0}-T_{b}}\right)*{\left(\frac{T_{m}-T}{T_{m}-T_{0}}\right)}^{\frac{T_{m}-T_{0}}{T_{0}-T_{b}}}\right)}^{a} \end{array}$$

where *r* is the calculated partial development rate, *T* denotes the hourly measured temperature, *T*_*b*_ denotes the minimal temperature for development, *T*_0_ denotes the optimal temperature for development (i.e., the highest development rate), *T*_*m*_ refers to the maximal temperature for development, and *a* denotes the shape of the slope (Figure 2); all parameters values determent during model optimization to data (Yin and Kropff, 1996).

The *r* -value was multiplied by the measured soil temperature and summed to estimate the effect of temperature on parasitism dynamics, where *R* is the accumulated GDDs:

(4) $$\begin{array}{l} R=\sum_{n=1}^{i}ri*Ti \end{array}$$

According to this model, the parasitism dynamics rate increases as the temperature rises from a minimal value to the optimal value. Beyond the optimal temperature, the parasitism dynamics rate decreases until the maximal temperature is reached. Temperatures below the minimal and beyond the maximal values did not contribute to the model and were therefore computed as zero.

#### Beta function model combined with a sigmoid curve (BTSG)

A third model was developed for prediction of *P. aegyptiaca* parasitism dynamics by combining the beta function Equation (3) with a sigmoid curve at the optimal temperature:

(5) $$\begin{array}{l} r=-\frac{1}{1+e^{\frac{-T+X_{0}}{b}}}+1 \end{array}$$

where *X*_0_ is the inflection point, *b* is the curve width, and *a* is the upper asymptote (in this case it is 1, the maximal rate). The sigmoid equation was combined with Equation (3) to develop Equation (6):

(6) $$\{\begin{array}{c} T<To\;\;r=[3]\\ T>To\;\;r=[5] \end{array}$$

**Figure 3:**
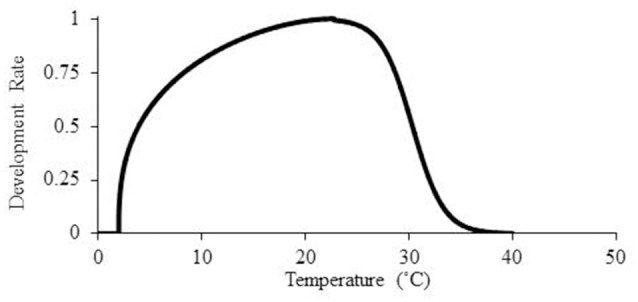
**Mathematical models for the description of ***P. aegyptiaca*** development rate as temperatures rise according to the beta function model combined with a sigmoid curve**.

According to Equation (6), the parasitism dynamics rate at high temperatures (above optimal temperature) will be calculated according to the sigmoid curve and not be assumed to be zero (Figure 3).

### *P. aegyptiaca* parasitism dynamic model

Partial development values were fitted to the different GDD models. For each model (linear, beta function and BTSG), a dynamic curve was fitted by four parameters (Weibull lag-phase equation):

(7) $$\begin{array}{l} f\left(x\right)=\left(1-\text{exp }\left(-{\left(\frac{GDD-lag}{b}\right)}^{c}\right)\right) \end{array}$$

where *f* (*x*) denotes the partial number of attachments out of the total number, *a* is the upper asymptote (in this case *a* = 100), *c* is defined as the slope of the curve, *b* is a scale parameter regardless of the shape value, *lag* is defined as the minimal GDD number for the appearance of attachments, and *GDD* is the calculated value on the *X*-axis (Ephrath and Eizenberg, 2010).

Curve fitting was performed according to root mean square error values (RMSE), where a RMSE-value represents the error between the observed and expected value, computed by:

(8) $$\begin{array}{l} RMSE=\sqrt{\frac{1}{n\sum_{i=1}^{n}{\left(x_{i}-y_{i}\right)}^{2}}} \end{array}$$

where *x*_*i*_ is the observed partial number of attachments number, *y*_*i*_ is the predicted number of attachments, and *n* is the number of observations. Lower RMSE-values indicate a better-fitted model (Lati et al., 2013).

A value that may be used for comparison of models is the Akaike information criterion correction (AICc), which is applied to take into consideration the model complexity and modeling accuracy according to the number of parameters in the model. In other words, AICc provides an estimate of the expected discrepancy between the model generating the data and a fitted candidate model (Cavanaugh, 1997). The AICc-value, with a normal distribution, is obtained by:

(9) $$\begin{array}{l} AICc=\;2m+n\text{ ln }\left(\frac{RSS}{n}\right)+\frac{2*m\left(m+1\right)}{n-m-1} \end{array}$$

where *m* is the number of model coefficients, *n* is the number of observations, and RSS is the residual sum of squares. A lower *AICc*-value indicates a better-fitted GDD prediction model (Lati et al., 2011).

### Validation of the models

To validate the different models, a leave-one-out cross validation procedure was conducted (Hawkins et al., 2003). By using cross validation, it is possible to use data as a learning group and a testing group simultaneously. For each specific computed model (beta function and BTSG), prediction was computed according to 12 out of 13 locations. The estimation of the one-out location was computed, following the learning group fitted parameters. The mean ± standard error for 12 locations was defined as the prediction area of the calculated model. A prediction was defined as true when the leave-one-out value lay in the prediction area (1), otherwise it was defined as false (0). The test was repeated for all 13 locations, using each location both as part of the learning group and as part of the validation group. The difference in days between the prediction and the observed result was found by matching the expected and the observed results to the calculated GDD from the data logger.

### Statistical analysis

The controlled environment experiments were arranged in a two-factorial design (temperature, day length) and repeated twice. ANOVA and comparison of the means were conducted by Tukey–Kramer HSD test (α < 0.05) using JMP software (vers. 7, SAS). Optimization of the parameters of models (3) and (6) (i.e., finding the parameters that give the lowest variance between locations) for the first of appearance broomrape was achieved by using a simulated annealing method (Aarts et al., 1997), since the large number of parameters in each equation [four in Equation (3) and seven in Equation (6)] and the large number of locations (13) did not enable a pure solution for optimization. This method is designed to avoid local optimum solution fixation and search forward until the optimal highest solution is found. Computing was performed with MATLAB (version 2009b, MathWorks, Natick, MA, USA). Lag-phase curve fitting was performed with Sigmaplot software (vers. 11, SPSS, Chicago, IL, USA). Leave-one-out cross validation was performed with MATLAB.

## Results

### Controlled-environment experiment

**Figure 4:**
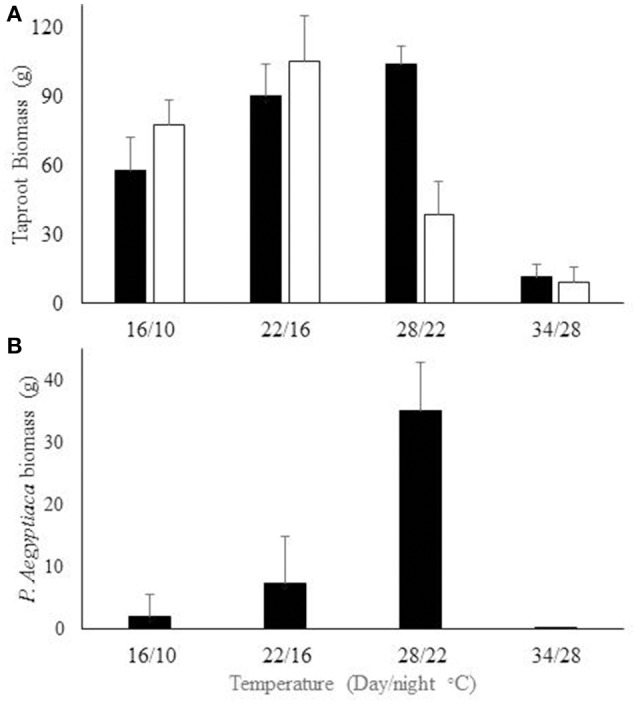
**(A)** Development of infested (□) and non-infested (■) carrot taproot biomass. **(B)** *P. aegyptiaca* biomass on carrot roots grown under different temperature regimes. Plants were grown in 2-L pots, two plants per pot. Bars indicate standard error, *n* = 5.

The effect of day length and temperature on carrot taproot and *P. aegyptiaca* development were examined under controlled-environment conditions. No mutual effect was found between temperature and day length. Temperature, however, did affect carrot biomass of both *P. aegyptiaca* infested and non-infested taproots. The maximal biomass of non-infested carrot taproots was obtained for a 28/22°C (day/night) temperature regime. However, maximal biomass of infested taproots was obtained for the 22/16°C (day/night) temperature regime. Maximal *P. aegyptiaca* biomass was obtained at 28/22°C (day/night). The lowest biomasses of carrot taproots (infested and non-infested) and of *P. aegyptiaca* were obtained at 34/28°C (day/night) (Figure 4).

### *P. aegyptiaca* field observations

The development of *P. aegyptiaca* was followed in 13 commercial carrot fields by using a minirhizotron camera. No correlation was found between the time after carrot seed planting and the appearance of the first *P. aegyptiaca* attachment on carrot roots (Table 1). Average temperatures at the different locations varied between 11 and 28°C, emphasis the temperature range (i.e. from maximum of 46°C to minimum of 0°C) in carrot growth as well as the temperature range that the model have to be valid.

### Models

Temperature data were converted to thermal time (GDD) using three different models: (a) linear model, (b) beta function model, and (c) BTSG model. The examined models were fitted to the same data set to identify the best approach for predicting *P. aegyptiaca* parasitism dynamics. The fitting process was done in three steps: (a) optimization of model parameters for first *P. aegyptiaca* appearance for minimal variance between locations, (b) dynamic curve fitting to the overall *P. aegyptiaca* development process, and (c) comparison of models and validation using a cross-validation procedure.

**Figure 5:**
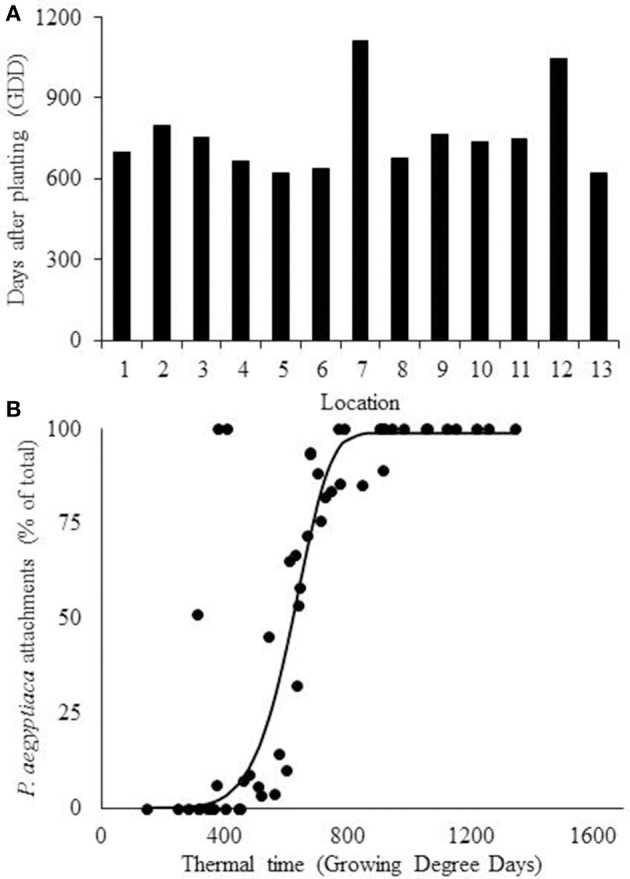
**(A)** Estimation of *Phelipanche aegyptiaca* parasitism with a linear model. **(A)** Detection of first *P. aegyptiaca* attachment according to the linear model using growing degree days (GDD). **(B)** *P. aegyptiaca* development dynamics according to the linear model. Curve parameters are presented in Table 3.

For prediction of the first *P. aegyptiaca* attachment, the linear model was fitted to the soil temperature data. The mean value (±*SD*) for the first *P. aegyptiaca* attachment was 759 ± 146 (*SD*) GDD (Table 2). The values for locations 7 and 12 were more than 2 *SD* units from the average value (Figure 5A). A Weibull lag-phase curve was fitted to data for demonstrating the parasitism dynamics, except for the higher asymptote (*a*), all other coefficients were not significant (Figure 5B, Table 3). Although, the model was found to be significant, with RMSE and AICc-values of 22.84 and 348.33, respectively, the model cannot predict *P. aegyptiaca* development dynamics due to the non-significance of its parameters.

**Table 2:** **Calculation of the appearance ***P. aegyptiaca*** first attachment as seen with the minirhizotron system, according to recorded soil temperature in 13 different locations**.

	**Mean**	**Variance**	**Standard deviation**
Linear	759.85	21, 409.30	146.31
Beta	656.19	3121.17	55.86
Beta-sigmoid	700.73	2890.03	53.75

*Model values were optimized to minimal variance by using Matlab*.

**Table 3:** **Non-linear four-parameter lag equation for describing the dynamics of ***P. aegyptiaca*** attachment on carrot roots according to the different models**.

**Method**	a^*^	***SE (a)***	***P (a)***	b^**^	***SE (b)***	***P (b)***	c^***^	***SE (c)***	***P (c)***	***LAG^+^***	***SE (LAG)***	***P (LAG)***	***P***	***R^2^***	***RMSE^++^***	***AICc^+++^***
Linear	98.66	5.61	<0.0001	653.99	1621.11	0.68	7.06	19.13	0.71	1.23^*^10^−13^	1617.29	1	<0.0001	0.73	22.84	348.33
Beta	96.75	4.72	<0.0001	85.61	20.96	0.0002	0.84	0.29	0.006	607.82	13.28	0.006	<0.0001	0.89	14.58	304.82
Beta–sigmoid	100	4.09	<0.0001	101.69	18.7	<0.0001	0.92	0.26	0.0009	583.83	13.64	<0.0001	<0.0001	0.94	10.79	278.58

*Curves were fitted with Sigmaplot software*.

* *Represents the maximal asymptote*.

** *The scale parameter regardless of the shape value*.

*** *Represents the shape parameter that determines the skewness and kurtosis of the equation*.

+ *Represents the lag phase until P. aegyptiaca attachment was initiated*.

++ *Root mean square error*.

+++ *Akaike Information Criterion (Corrected)*.

**Figure 6:**
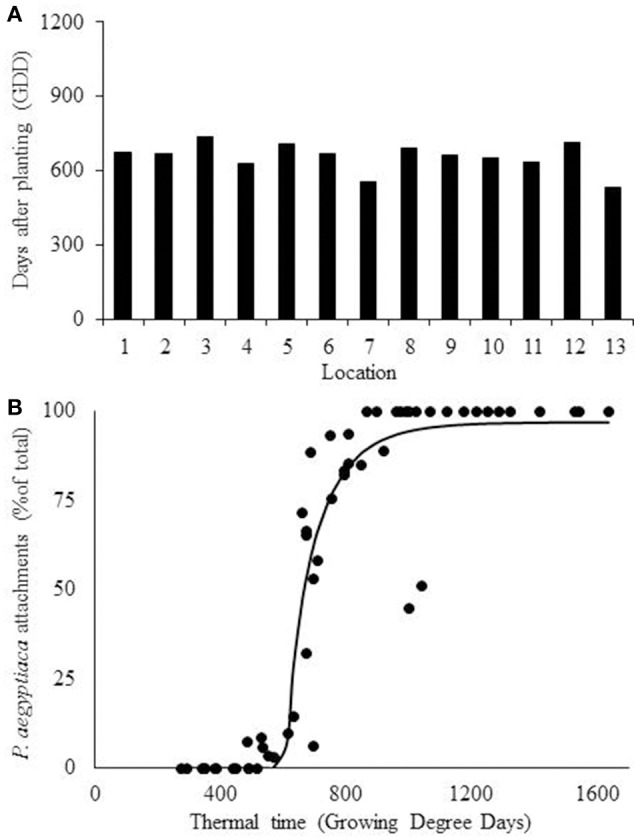
**Estimation of ***Phelipanche aegyptiaca*** parasitism according to the beta function model. (A)** Calculation of first *P. aegyptiaca* attachment according to the beta model. **(B)** *P. aegyptiaca* development dynamics according to the beta model. Observation were made in 13 locations. Curve parameters are presented in Table 3.

Fitting of the beta-function curve to the data set showed that the development rate was inhibited after the optimum point had been reached and stopped after the maximal temperature (Equation 3). According to the model, *P. aegyptiaca* first attachment was observed at 656 ± 55 (*SD*) GDD (Table 2, Figure 6A). A four-parameter Weibull equation with lag phase adoption curve was fitted to the beta function adjusted values (Figure 6B, Table 3). The first *P. aegyptiaca* attachment was calculated to occur after 607 GDD; the RMSE-value was 14.58, while the AICc-value was 304.82, i.e., the values were lower than those for the linear model.

**Figure 7:**
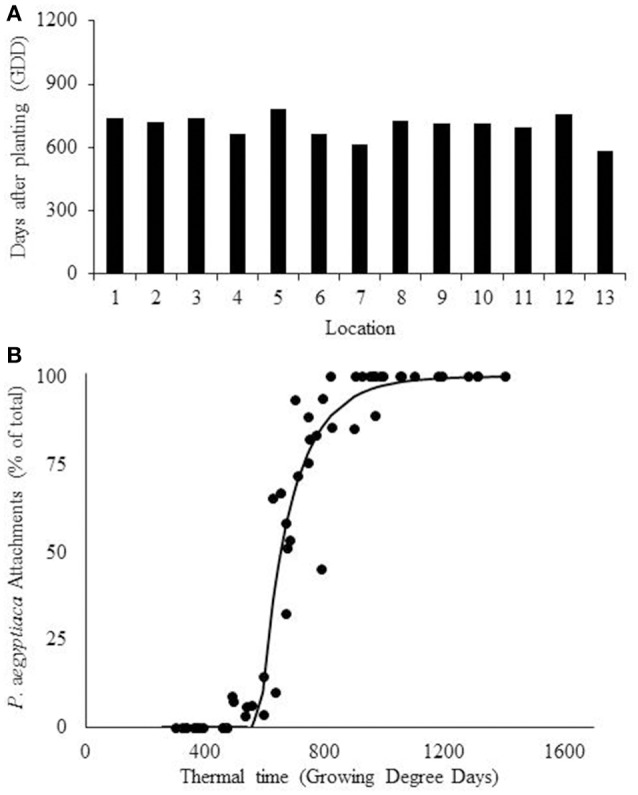
**Estimation of ***Phelipanche aegyptiaca*** parasitism with the combined beta function and sigmoid model. (A)** Calculation of first *P. aegyptiaca* attachment calculated according to the combined model. **(B)** *P. aegyptiaca* development dynamic according to the combined model, Observations in 13 locations. Curve parameters are presented in Table 3.

The BTSG model (Equation 6) was also fitted to the data (Figure 7B, Table 3). This model estimated that development does not cease after the maximal temperature, but decreases until it becomes asymptotically close to zero at higher temperatures. Fitting the model of the first *P. aegyptiaca* attachment for minimal variance gave a mean value (± *SD*) of 700 ± 53 GDD (Table 2, Figure 7A). In the second step, as was done for the other models, a dynamic curve was fitted to the *P. aegyptiaca* development observations. The first *P. aegyptiaca* attachments appeared after 583 GDD. The RMSE-value was 10.79, while the AICc-value was 278.58, both being lower than the values obtained with the other two models. For both beta function and BTSG models, data was calculated in two ways: hourly measurements and daily average. While hourly measurements resulted in good estimation with low variance, daily average measurements demonstrated poor estimation with high variance between locations. Therefore, further analyses of the models were done using hourly measurements.

### Comparison and field validation of the models' prediction power

The two best models were compared; the beta function and the BTSG models. These two models were validated for prediction of *P. aegyptiaca* first attachment by the leave-one-out cross validation method. Both models were able to predict *P. aegyptiaca* parasitism in all 13 locations (Tables 4, 5). The average variances of the beta function and the BTSG were 6228 and 5801, respectively. The average differences between the expected first *P. aegyptiaca* attachment and the observed attachment were 2.16 and 2.35 days for the beta function and the combined models, respectively. The maximal time gap between the observed *P. aegyptiaca* first attachment and the prediction made by the model were 5.13 and 5 days for the beta function and the BTSG models, respectively.

**Table 4:** **Leave-one-out cross validation for prediction of first ***P. aegyptiaca*** attachment on carrot roots according to beta-function-calculated subsoil temperatures**.

**Site out number**	**Mean**	**One-out value**	**Variance**	***SD***	**Yes/No**	**Difference**	**Days**
1	689.75	708.48	6145.69	78.39	1	18.73	1.00
2	694.95	683.09	6183.63	78.63	1	11.86	1.25
3	679.12	749.84	6276.35	79.22	1	70.72	3.17
4	700.41	652.74	6027.26	77.63	1	47.67	3.75
5	690.13	727.74	6370.49	79.81	1	37.61	1.33
6	711.01	678.6	6178.69	78.6	1	32.41	1.79
7	694.16	692.82	6115.01	78.19	1	1.34	0.83
8	690.49	717.12	6314.86	79.46	1	26.63	1.33
9	692.86	677.78	6269.45	79.17	1	15.08	1.25
10	695.99	663.18	6332.43	79.57	1	32.81	5.13
11	694.25	642.06	5950.98	77.14	1	52.19	5.08
12	700.94	682.79	6266.83	79.16	1	18.15	1.21
13	689.56	711.78	6535.71	80.48	1	22.22	1.00
Average	694.12	691.38	6228.26	78.88	1	29.80	2.16

*Means and SD were computed with 12 sites, while the site out value was computed by the estimated parameters. Difference refers to calculated GDDs gap between the mean and the one-out value. Days represent the difference converted into days*.

**Table 5:** **Leave-one-out cross validation for prediction of first ***P. aegyptiaca*** attachment on carrot roots according to beta function combined with sigmoid equation calculated sub soil temperatures**.

**Site-out number**	**Mean**	**One-out value**	**Variance**	***SD***	**Yes/No**	**Difference**	**Days**
1	702.74	729.37	5852.42	76.50	1	26.63	1.83
2	709.71	741.68	5855.55	76.71	1	31.97	2.29
3	691.15	751.16	5721.73	75.64	1	60.01	2.92
4	709.07	666.9	5754.78	75.86	1	42.17	2.96
5	698.29	767.48	5567.25	74.61	1	69.19	5.00
6	726.48	679.24	5807.28	76.20	1	47.24	2.50
7	703.96	669.74	5834.79	76.38	1	34.22	4.22
8	698.9	719.77	5859.39	76.54	1	20.87	1.21
9	704.18	716.76	5866.98	76.59	1	12.58	1.00
10	707.05	723.31	5868.64	76.60	1	16.26	2.04
11	707.32	709.62	5879.59	76.67	1	2.3	0.21
12	704.31	630.88	5697.78	75.48	1	73.43	4.21
13	703.88	701.71	5855.47	76.52	1	2.17	0.17
Average	705.15	708.27	5801.66	76.17	1	33.77	2.35

*Means and SD were computed with 12 sites, while the site out value was computed by the estimated parameters. Difference refers to calculated gap in GDDs between the mean and the one-out value. Days represent the difference converted into days*.

## Discussion

Most weed species develop above the soil surface and can therefore be observed and quantified (e.g., weed height or biomass). However, in the case of root parasitic weed, broomrape, development, and biomass accumulation take place in the soil sub-surface and are therefore extremely difficult to quantify. Several methods have been developed for broomrape detection. One of the most promising relies on thermal time or GDD models. These models assume a positive linear correlation between soil temperatures and broomrape development (Eizenberg et al., 2012a). However, the current study found that *P. aegyptiaca* parasitism on carrot roots responded differently from that reported in previous studies, in which low temperatures (below 18°C) were found to be essential for *P. aegyptiaca* and *O. crenata* development on carrot roots (Eizenberg et al., 2001). In the current work, results from the controlled-environment experiments demonstrated that the *P. aegyptiaca* development rate on carrot roots accelerated until an optimal temperature (28°C/22°C day/night) was reached; at higher temperatures *P. aegyptiaca* development was inhibited.

The controlled-environment experiments on *P. aegyptiaca*–infested carrot plants demonstrated that the optimal temperatures for parasite and carrot development were similar, being 28°C/22°C and 22°C/16°C, respectively, for *P. aegyptiaca* infested carrot taproots. At high temperatures development of both *P. aegyptiaca* and carrot taproot was inhibited. In previous research, a linear model was found to adequately describe the relations between thermal time and parasitism dynamics for *O. minor* on red clover, *O. cumana* on sunflower, and *P. aegyptiaca* on tomato (Eizenberg et al., 2005a, 2012b; Ephrath et al., 2012). However, model fitting to the current data using the linear model resulted in poor prediction. Assessment of first *P. aegyptiaca* attachment by using the linear model resulted in high variation, i.e., 2 out of the 13 locations gave higher values than the average predicted by the linear model. Moreover, the equation describing the parasitism dynamics failed to describe the first appearance of *P. aegyptiaca* attachment according to the linear model [the parameter lag phase on the Weibull Equation (7)]. The controlled-environment experiment confirmed that carrot optimal growth rate rise until 28°C/22°C, and thereafter carrot growth decreases above the optimal temperature. It has been reported that *P. aegyptiaca* seed germination is also inhibited at high temperatures (Kebreab and Murdoch, 1999). According to these results, a linear model cannot describe the parasitism dynamics. Thus, these findings emphasize the need for a different model for prediction at temperatures beyond the optimal values.

The beta function model addresses the above problem by adding the maximal temperature for development parameter (*T*_*m*_). According to the model, development begins above the minimal temperature (*T*_*b*_), rises until the optimal temperature (*T*_*o*_), decreases between the optimal and the maximal temperatures, and ceases completely beyond *Tm* (Yin and Kropff, 1996). Using the beta function model, we were able to reduce the variance in the first appearance of *P. aegyptiaca* between the different locations, compared to the linear model. The minimal temperature for development (*T*_*base*_), according to the fitted model, was found to be 2°C, as was previously reported for the development of carrot seeds and seedlings (Finch-Savage et al., 1998). This *T*_*base*_ was appropriate for carrots but for not *P. aegyptiaca* germination, which was found to be 4–5°C (Murdoch and Kebreab, 2013). This disparity can be explained by the understanding that the model refers to overall parasitism dynamics, which include *P. aegyptiaca* that is parasitized carrot. Therefore, we can assume that seed germination, a short part of the attachment process, plays a negligible role in the parasitism dynamics. Moreover, the effect of temperature on host development is more important than its effect on the parasite, because the germination of *P. aegyptiaca* seeds is autonomic, while its development (i.e., biomass accumulation) is dependent on the host conditions; therefore, the effect of soil temperature on host development is more significant for *P. aegyptiaca* biomass accumulation than for its germination.

Previous research has shown different *P. aegyptiaca* base temperatures for development on different crops (Ephrath and Eizenberg, 2010; Ephrath et al., 2012). Unlike the linear model, the dynamics curve according to the beta function can predict the entire parasitism progression. However, it cannot be assumed that there is complete inhibition of parasite development beyond the optimal temperature. Moreover, although previous research has described optimal parasite germination at temperatures up to 30°C, parasite germination is still possible above this temperature (Murdoch and Kebreab, 2013). In addition, parasite genetic diversity should be taken into consideration, and therefore we cannot assume unequivocally that no parasite development has occurred above the maximal temperature. Accordingly, an adjusted model was developed for use at temperatures above the optimal temperature, namely, a beta function model combined with a sigmoid curve, the BSTG model. According to the BTSG model, beyond the optimal temperature there is a moderate decline of the development rate, instead of a rapid decline to zero. This model describes non-symmetric development under rising temperatures. As demonstrated in the controlled environment experiment and in the field minirhizotron observations, development at low temperatures differs from that at high temperatures. Therefore, the BTSG model—with a particular development rate up to the optimal temperature and a different rate beyond it—can provide a good estimation of parasitism dynamics.

Cross validation analysis of both models confirmed the utility of the BTSG model. The BTSG model and the beta function model made similar predictions for the first *P. aegyptiaca* attachment, but prediction of overall *P. aegyptiaca* parasitism dynamics was found to be superior with the combined model. RMSE- and AICc-values confirm the robustness of using the combined model, even though it contains more parameters.

Although chemical control is a practical tool for broomrape management (Eizenberg et al., 2012a), effective use of herbicides requires accurate application at a specific parasite development stage; for example, for red clover infected by *O. minor*, imazamox application to the foliage according to thermal time model reduced the damage caused to crop by the parasite. Moreover, herbicide application at an early parasite developmental stage has the dual advantage of requiring a lower dose of the chemical than that at a later stage and remaining effective for a longer period (Eizenberg et al., 2006). Therefore, accurate prediction of initiation of *P. aegyptiaca* attachment is a prerequisite for optimal herbicide application (Cochavi et al., 2016). However, field experiments have shown that the best results are obtained using sequential herbicide application (Cochavi et al., 2015), and it is therefore essential to have in hand a parasitism dynamics model for accurately predicting the timing of herbicide application. Previously developed models are not suitable, as they pertain to short-term crops grown over the summer or greenhouse crops grown mostly under moderately high temperature regimes.

Carrot was originally a moderate temperature crop, but nowadays it is grown the year round. Therefore, the crop is exposed to a wide range of temperatures, including supra optimal temperatures, reducing its development rate during the growth period. It is well-known from studies on non-parasitic weeds that the development rate at high temperatures is not linear and tends to decline (White et al., 2015). Hence, the prediction model should deal differently with the crop–parasite relation at different temperatures. The beta-function model, unlike the linear model, gives an accurate prediction of the first establishment of *P. aegyptiaca* on carrot roots, due to its ability to deal with the parasite development at supra-optimal temperatures. However, it is known from previous research that beneficial *P. aegyptiaca* management in the field requires at least three sequential applications of glyphosate, not only at the first attachment but also at later stages (Cochavi et al., 2015). Therefore, although the BTSG model contains more parameters than the beta function model and can predict the *P. aegyptiaca* first attachment at the same level, its ability to predict more accurately the total parasitism process is superior, as is needed for optimal herbicide application in the field.

## Conclusions

A number of models have been developed to describe the parasitism dynamics of broomrape species in their hosts, and in all a linear equation for computing GDD was fit to the parasitism dynamics. The point to be stressed is that all these models are seasonal and quantify the parasitism dynamics in the spring when temperatures begin to increase. However, carrot are cultivated the year round, with sowing in the summer and growth into the winter or be sowing in the winter and growth into the summer. Therefore, for this host, it was necessary to develop a robust temperature-sensitive model for predicting the parasitism dynamics. Hence, the BTSG model was found to be effective for prediction of *P. aegyptiaca* development in all temperature regimes, including high temperatures. The model found to be effective for predicting the entire parasitism dynamics and not only for predicting the first attachment therefore can be used for precise temporal chemical management of in carrot.

Further research should test herbicide efficiency according to the suggested model, meaning the best timing of applications and the optimal number of applications for the development of a sustainable method for *P. aegyptiaca* management in carrot.

## Author contributions

AC: MSc student, performed field studies, data analysis, and writing the manuscript. GA: Research Assistant, technical support in field studies. BR: Supervisor, support in data analysis and writing. HE: Principle investigator, head of the lab of parasitic weeds.

### Conflict of interest statement

The authors declare that the research was conducted in the absence of any commercial or financial relationships that could be construed as a potential conflict of interest.
